# Radiographic Progression-Free Survival and Clinical Progression-Free Survival as Potential Surrogates for Overall Survival in Men With Metastatic Hormone-Sensitive Prostate Cancer

**DOI:** 10.1200/JCO.23.01535

**Published:** 2024-01-05

**Authors:** Susan Halabi, Akash Roy, Larysa Rydzewska, Siyuan Guo, Peter Godolphin, Maha Hussain, Catherine Tangen, Ian Thompson, Wanling Xie, Michael A. Carducci, Matthew R. Smith, Michael J. Morris, Gwenaelle Gravis, David P. Dearnaley, Paul Verhagen, Takayuki Goto, Nick James, Marc E. Buyse, Jayne F. Tierney, Christopher Sweeney

**Affiliations:** ^1^ Duke University Medical Center, Duke University, Durham, NC; ^2^ Medical Research Council Clinical Trials Unit at University College London, London, United Kingdom; ^3^ Robert H. Lurie Comprehensive Cancer Center, Northwestern University, Chicago, IL; ^4^ Fred Hutchinson Cancer Research Center, Seattle, WA; ^5^ Christus Health, San Antonio, TX; ^6^ Dana-Farber Cancer Institute, Boston, MA; ^7^ Sidney Kimmel Cancer Center, Johns Hopkins University, Baltimore, MD; ^8^ Massachusetts General Hospital Cancer Center, Boston, MA; ^9^ Division of Solid Tumor Oncology, Memorial Sloan Kettering Cancer Center, New York, NY; ^10^ Institut Paoli-Calmettes Aix-Mareseille Université, Marseille, France; ^11^ Institute of Cancer Research, The Royal Marsden NHS Foundation Trust, London, United Kingdom; ^12^ Erasmus Medical Center, Rotterdam, the Netherlands; ^13^ Department of Urology, Graduate School of Medicine, Kyoto University, Kyoto, Japan; ^14^ International Drug Development Institute, Louvain-La-Neuve, Belgium; ^15^ University of Adelaide, Adelaide, Australia

## Abstract

**PURPOSE:**

Despite major increases in the longevity of men with metastatic hormone-sensitive prostate cancer (mHSPC), most men still die of prostate cancer. Phase III trials assessing new therapies in mHSPC with overall survival (OS) as the primary end point will take approximately a decade to complete. We investigated whether radiographic progression-free survival (rPFS) and clinical PFS (cPFS) are valid surrogates for OS in men with mHSPC and could potentially be used to expedite future phase III clinical trials.

**METHODS:**

We obtained individual patient data (IPD) from 9 eligible randomized trials comparing treatment regimens (different androgen deprivation therapy [ADT] strategies or ADT plus docetaxel in the control or research arms) in mHSPC. rPFS was defined as the time from random assignment to radiographic progression or death from any cause whichever occurred first; cPFS was defined as the time from random assignment to the date of radiographic progression, symptoms, initiation of new treatment, or death, whichever occurred first. We implemented a two-stage meta-analytic validation model where conditions of patient-level and trial-level surrogacy had to be met. We then computed the surrogate threshold effect (STE).

**RESULTS:**

IPD from 6,390 patients randomly assigned from 1994 to 2012 from 13 units were pooled for a stratified analysis. The median OS, rPFS, and cPFS were 4.3 (95% CI, 4.2 to 4.5), 2.4 (95% CI, 2.3 to 2.5), and 2.3 years (95% CI, 2.2 to 2.4), respectively. The STEs were 0.80 and 0.81 for rPFS and cPFS end points, respectively.

**CONCLUSION:**

Both rPFS and cPFS appear to be promising surrogate end points for OS. The STE of 0.80 or higher makes it viable for either rPFS or cPFS to be used as the primary end point that is surrogate for OS in phase III mHSPC trials with testosterone suppression alone as the backbone therapy and would expedite trial conduct.

## INTRODUCTION

Metastatic prostate cancer is an incurable disease which ultimately claims the lives of about 27,000 American men each year.^1^ For decades, the only available treatment for men with metastatic hormone-sensitive prostate cancer (mHSPC) has been life-long hormonal therapy with the backbone of therapy being testosterone suppression (TS) alone, also commonly known as androgen deprivation therapy (ADT).^2^ The landscape in mHSPC underwent a major shift when the CHAARTED^3^ and docetaxel arm of STAMPEDE^4^ trials showed a significant overall survival (OS) benefit from the concurrent administration of ADT plus docetaxel, confirmed by a subsequent systematic review. However, not all patients benefited from chemotherapy,^3,5^ and some patients experienced toxicity without benefit. Specifically, CHAARTED men with high-volume disease had a clearer survival benefit in long-term follow-up compared with men with low volume metastatic disease. Recent trials demonstrated OS advantage in men with mHSPC treated with ADT and novel hormonal therapies.^6,7^

**CONTEXT**

**Key Objective**
Overall survival (OS) is considered as the gold standard end point in phase III trials. However, given the outcomes in men with metastatic hormone-sensitive prostate cancer (mHSPC) treated with current therapy, it takes close to a decade to complete new mHSPC studies with OS as the primary end point. Our primary objective was to evaluate if radiographic progression-free survival (rPFS) and clinical PFS (cPFS) are valid surrogates for OS and could potentially be used to speed phase III clinical trials.
**Knowledge Generated**
Both rPFS and cPFS appear to be promising surrogate end points for OS that could be used as end points in future trials in men with mHSPC.
**Relevance *(G.K. Schwartz)***
The use of rPFS and cPFS as valid surrogate end-points for OS should accelerate drug approval in the treatment of patients of mHSPC.*
  *Relevance section written by *JCO* Associate Editor Gary K. Schwartz, MD, FASCO.

Despite major increases in the longevity of men with mHSPC, most men who have the disease still die of prostate cancer. Thus, a major unmet need in mHSPC is for the development of novel therapies for these men. Approval of new therapies will be granted on the basis of well-powered phase III trials with solid end points. One of the most important factors in the design of a clinical trial is the choice of the primary end point that will determine the sample size and trial duration. Although OS remains the gold standard end point in phase III mHSPC trials, given the OS outcomes with current therapy of ADT plus newer hormonal therapies, it will take close to a decade to complete new mHSPC studies with OS as the primary end point. Although OS is clearly defined, simple to measure, and translates into clinical benefit to patients, it has the disadvantages of requiring large trial sizes and an extended follow-up period leading to long study durations in mHSPC trials.

Reflecting this, there is widespread interest among clinical investigators, sponsors, and regulators in using intermediate clinical end points (ICEs) to help make decisions about the efficacy of certain drugs or biologic devices by means of well-powered comparative trials. Such end points are potentially more readily available earlier in the course of the cancer's natural history, are measured more frequently, are less costly, and thus might be more appropriate than OS.^8-10^ However, before an ICE can replace OS, it needs to be formally validated. Current state-of-the-art validation methodology uses a correlation approach based on a two-stage model.^11,12^ Within this framework, a surrogate may be assessed both at the trial level and the individual level for its ability to predict the effect of treatment on OS.

We hypothesized that radiographic progression-free survival (rPFS) and clinical PFS (cPFS) are valid surrogates for OS in men with mHSPC and could potentially be used to expedite phase III clinical trials. We also evaluated the surrogacy in subgroups of patients on the basis of treatment received, high-volume disease, and de novo metastatic diagnosis.

## METHODS

### Search Strategy and Selection Criteria

We identified eligible trials and searched electronic databases from Medline, Embase, clinical trials registers (ClinicalTrials.gov and the Cochrane Central Register of Controlled Trials) proceedings and abstracts of relevant conferences and followed the preferred reporting items for systematic reviews and meta-analyses (PRISMA) guidelines. The inclusion criteria listed randomized clinical trials phase II/phase III trials that completed accrual and follow-up and accrual was after January 1992. We excluded trials where the primary end point was safety, quality of life, and feasibility. At the time of the initial project in 2018, we identified 51 mHSPC trials (14,898 patients). Individual patient data (IPD) from nine eligible randomized trials (13 comparisons) that compared treatment regimens (ADT or ADT plus docetaxel in the control or research arms) in mHSPC were used.^3-5,13-20^ The STAMPEDE was a multiarm platform trial where controls were shared across the comparisons. Controls were randomly split 100 times by the two periods: (2005-2011) or (2011-2013) and were made independent across the five comparisons to minimize the bias in estimating the parameters of interest (hazard ratios [HRs], *R*^2^, and surrogate threshold effect [STE]; Data Supplement, online only).

### Definition of End Points

The established end point is OS, which was defined as the interval from the date of random assignment to the date of death from any cause; if patients had not died, they were censored at the date of the last follow-up. rPFS was defined as the time from random assignment to radiographic progression (defined per protocol) or death from any cause, whichever occurred first; cPFS was defined as the time from random assignment to the date of radiographic progression, symptoms, initiation of a new treatment, or death, whichever occurred first.

### Data Analysis

#### Surrogacy Criteria

We evaluated the surrogacy of the ICEs (rPFS and cPFS) with OS by using the standard meta-analytic two-stage validation model.^11,12^ A description of the two-stage modeling is presented in the Data Supplement. In brief, in the first stage, we tested for individual-level association between the ICEs and OS and computed the trial-specific effects of treatment of the ICE and OS. In the second stage, weighted linear models (WLRs) were used to test for the correlation between the treatment effects on both the ICEs and OS. We defined a priori a clinically relevant surrogacy of *R*^2^ value >0.75, which is considered a standard surrogacy assessment in oncology.^8^ The primary analysis was based on the 13 comparisons (nine trials). Secondary analyses excluded trials with insufficient follow-up where the median follow-up was <4.3 years (CALGB 90202, ZAPCA, and HOG). Condition 1 was tested at the individual patient-level and the trial-level data. Patient-level associations of OS with rPFS and cPFS were evaluated using the bivariate copula models on IPD accounting for trial-specific treatment effects on rPFS, cPFS, and OS.^11,12^ The Weibull distribution was assumed to evaluate the effect of treatment on the marginal distribution of each end point. The Clayton copula was chosen for both ICEs as it provided the best model fitness on the basis of the regularized goodness-of-fit tests.

At the trial-level analysis, Kaplan-Meier (KM) estimates of 3-year ICEs rates and 5-year OS rates for each treatment arm were computed. These time points were chosen because of the fact that 3-year ICEs data would expedite clinical trial readout, whereas 5-year OS rate is beyond the observed median OS of the mHSPC trials included in the database with TS alone as the hormonal therapy backbone and would be considered mature follow-up. We performed WLR analyses on the basis of the inverse variance of the OS estimates stratified by trial and treatment arm. *R*^2^ was used to quantify the proportion of variance that was explained by the regressions.

For condition 2, we used the proportional hazards (PH) model to obtain study-specific treatment effects, that is, log(HRs) of the ICEs and OS. The PH assumption was checked for each of the 13 units separately. WLR incorporated the effects of treatment on OS versus the effects of treatment on ICEs, where weights were the inverse variances of the natural log(HR) of the OS. Model accuracy was assessed by the leave-one-out-cross validation (LOOCV) procedure. Subgroup analyses were performed on the basis of the type of primary therapy received, volume of disease (high, low), and M1 diagnosis status, although we did not report results for patients with metachronous disease because of the small sample size. Finally, we defined the STE as the intersection of the horizontal line at log(HR) = 0 for OS with the upper 95% prediction limit for the regression line of the effect of treatment on OS versus the effect of treatment on the ICEs.^9^

## RESULTS

**FIG 1.**
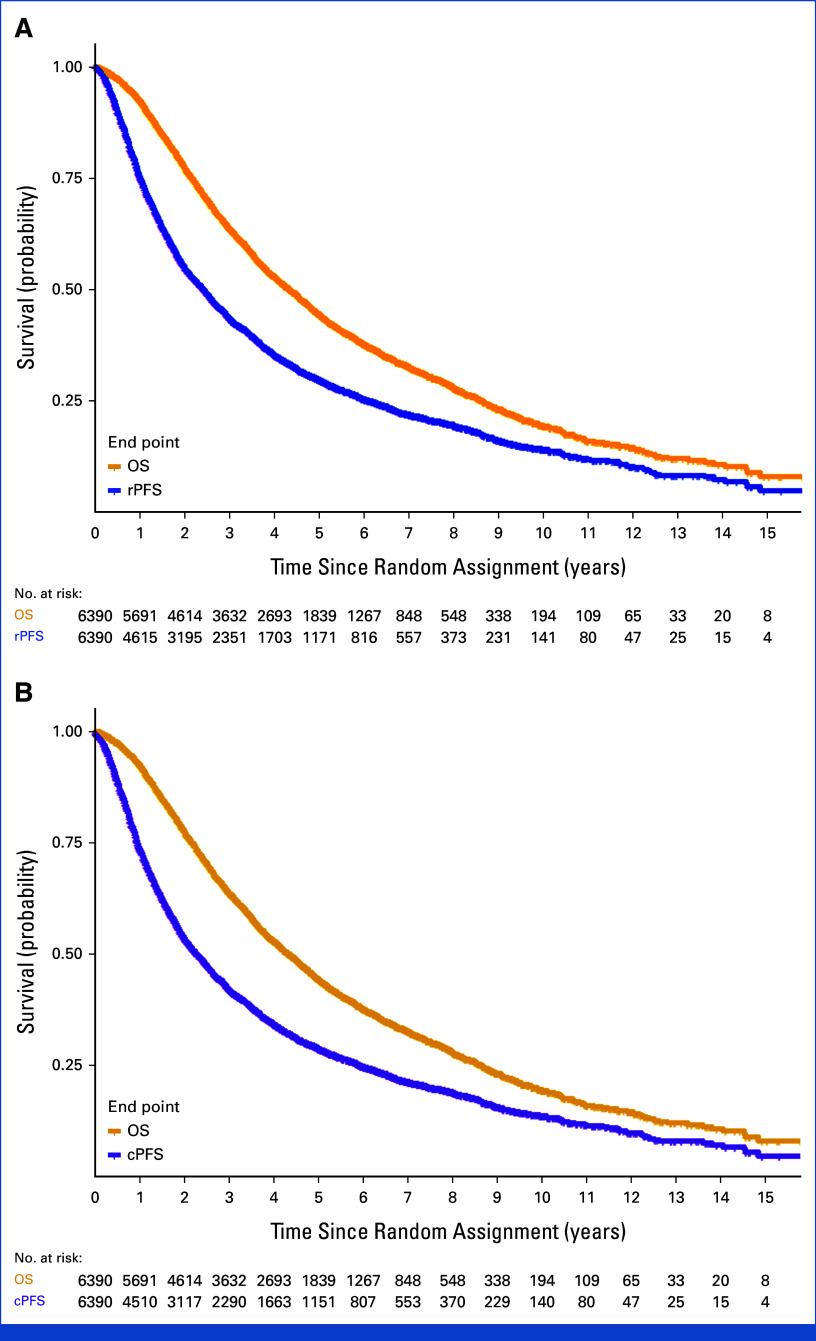
Kaplan-Meier curves for (A) OS and rPFS and (B) OS and cPFS. cPFS, clinical progression-free survival; OS, overall survival; rPFS, radiographic progression-free survival.

Thirteen comparisons were available with a total of 6,390 men with HSPC. Patients were enrolled in these trials from June 1994 to July 2013 (Data Supplement, Table S1 and Fig S1). The median age was 67 years, 70% of patients had Eastern Cooperative Oncology Group performance status of 0, and 77% had de novo diagnosis (Table 1). Thirty-six percent of patients had high-volume disease and had missing data on this factor. The KM OS curves for OS and the ICEs are presented in Figures 1A and 1B. The estimated hazard functions for OS and the ICEs are presented in the Data Supplement (Figs S2A and S2B). About 71% of men had died of PC, and the median follow-up in 2,529 surviving patients was 6.1 years (range, 0.0-17.7). There were a total of 4,501 rPFS (55% are radiographic progression, 45% were deaths) and 4,574 cPFS events. The median OS was 4.3 years (95% CI, 4.2 to 4.5), whereas the median rPFS and cPFS were 2.4 and 2.3 years, respectively.

**TABLE 1.** Baseline Characteristics of 6,390 Men With mHSPC

Characteristic	N = 6,390
Age at random assignment, years, median (IQR)	67 (61.0-73.1)
Age at random assignment, No. (%)	
Younger than 65	2,575 (40.3)
65-70 years	1,366 (21.4)
70-75 years	1,212 (19.0)
75-79 years	794 (12.4)
Older than 79	443 (6.9)
Race, No. (%)	
Asian and other	378 (5.9)
Black	269 (4.2)
White	2,264 (35.4)
Unknown/missing	3,479 (54.4)
Year of random assignment, No. (%)	
1994-1998	439 (6.9)
1999-2003	846 (13.2)
2004-2008	2,064 (32.3)
2009-2013	2,244 (35.1)
Missing	797 (12.5)
Type of therapy used,^a^ No. (%)	
ADT plus docetaxel	2,627 (41.1)
ADT alone	4,487 (70.2)
Performance status, No. (%)	
0	4,451 (69.7)
1	1,708 (26.7)
2	178 (2.8)
3	1 (0.0)
Missing	52 (0.8)
Volume of disease, No. (%)	
Low	1,789 (28.0)
High	2,297 (35.9)
Unknown	2,304 (36.1)
M1 diagnosis status, No. (%)	
Synchronous	4,891 (76.5)
Metachronous	1,068 (16.7)
Unknown	431 (6.8)
Gleason score, No. (%)	
<4	16 (0.2)
4-6	493 (7.7)
7	1,487 (23.2)
8-10	3,405 (53.3)
Missing	989 (15.5)
Prior treatment, No. (%)	
No treatment	2,369 (37.1)
Radical prostatectomy	572 (9.0)
Missing	3,449 (54.0)
Radiation therapy, No. (%)	
No treatment	2,257 (35.3)
Radiation therapy	613 (9.6)
Missing	3,520 (55.1)
Laboratory	
PSA, ng/L, median (IQR)	20.7 (1.0-135.0)
Missing, No. (%)	78 (1.2)
HgB, g/dL, median (IQR)	13.7 (12.6-14.6)
Missing, No. (%)	2,711 (42.4)
Alkaline phosphatase, U/L, median (IQR)	126.0 (78.0-310)
Missing, No. (%)	2,653 (41.5)
Testosterone, ng/mL, median (IQR)	2.6 (0.4-4.6)
Missing, No. (%)	5,029 (78.7)
Albumin, g/dL median (IQR)	42.0 (39.0-45.0)
Missing, No. (%)	3,997 (62.6)
LDH, U/L, median (IQR)	181.0 (154.2-225)
Missing, No. (%)	5,532 (86.6)

Abbreviations: ADT, androgen deprivation therapy; LDH, lactate dehydrogenase; PSA, prostate-specific antigen.

a The STAMPEDE ADT arms are not mutually exclusive since they have shared controls.

### Surrogacy Analysis: Overall

#### Condition 1

**FIG 2.**
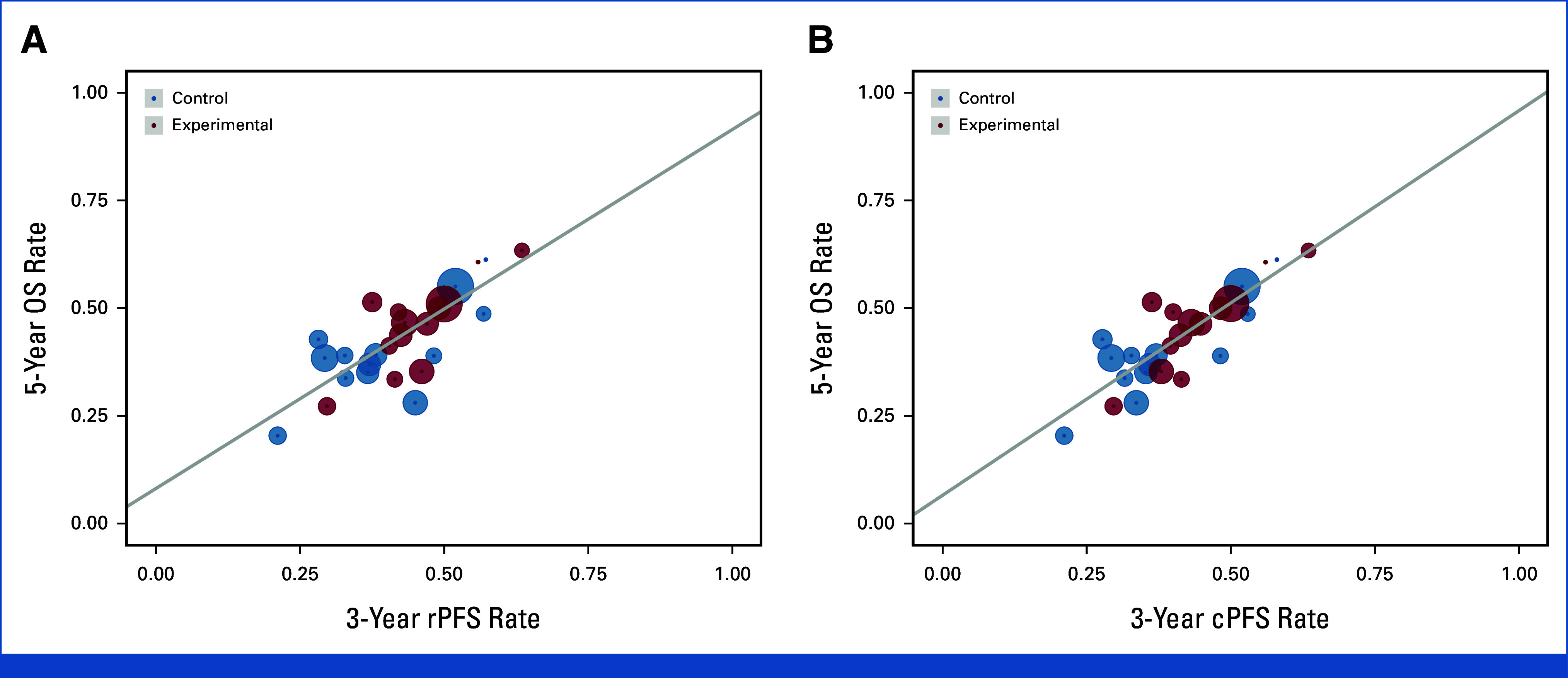
5-year OS rate versus 3-year ICEs rates: (A) 5-year OS versus 3-year rPFS and (B) 5-year OS versus 3-year cPFS. All rates were estimated by the Kaplan-Meier stratified by trial and treatment arm. Circle size was the sample size of each trial and regression was weighed by the inverse variance of the 5-year OS rate estimates. cPFS, clinical progression-free survival; ICEs, intermediate clinical end points; OS, overall survival; rPFS, radiographic progression-free survival.

At the patient level, Kendall's tau was 0.83 (95% CI, 0.82 to 0.84; Table 2) for rPFS and OS and 0.85 (95% CI, 0.85 to 0.86) for cPFS and OS. The KM OS rates at 5- versus 3-year ICEs stratified by treatment arm and trial are presented in Figures 2A and 2B. From WLR, *R*^2^ between 3-year rPFS and 5-year OS rates and 3-year cPFS rates was 0.62 (95% CI, 0.29 to 0.89) and 0.74 (95% CI, 0.49 to 0.90), respectively. When limiting the analysis to patients with sufficient follow-up, *R*^2^ between 3-year rPFS and 5-year OS rates was 0.74 (95% CI, 0.40 to 0.96).

**TABLE 2.** Summary of the Results From the Two-Stage Surrogacy Analysis

Two-Stage Meta-Analytic Validation Model	No. of Units (No. of patients)	Condition 1: ICEs and OS Are Correlated		Condition 2: Treatment Effects on Both End Points Are Correlated	Regression Equation
End point		Correlation at the Patient Level, Kendall's Tau (95% CI)	Regression of 5-Year OS Rate *v* 3-Year ICE Rate by Trial and Arm Weighted by the Inverse Variances of OS, *R*^2^ (95% CI)	Regression of log(HR)-OS *v* log(HR)-ICE by Trial Weighted by the Inverse Variances of OS, *R*^2^ (95% CI)	
rPFS	13 (6,390)	0.83 (0.82 to 0.84)	0.62 (0.29 to 0.89)	0.83 (0.64 to 0.98)	log OS (HR) = 0.004 + 0.798 × log rPFS (HR)
cPFS	13 (6,390)	0.85 (0.85 to 0.86)	0.74 (0.49 to 0.90)	0.84 (0.61 to 0.99)	log OS (HR) = 0.002 + 0.797 × log cPFS (HR)

Abbreviations: cPFS, clinical progression-free survival; HR, hazard ratio; ICEs, intermediate clinical end points; OS, overall survival; rPFS, radiographic progression-free survival.

#### Condition 2

**FIG 3.**
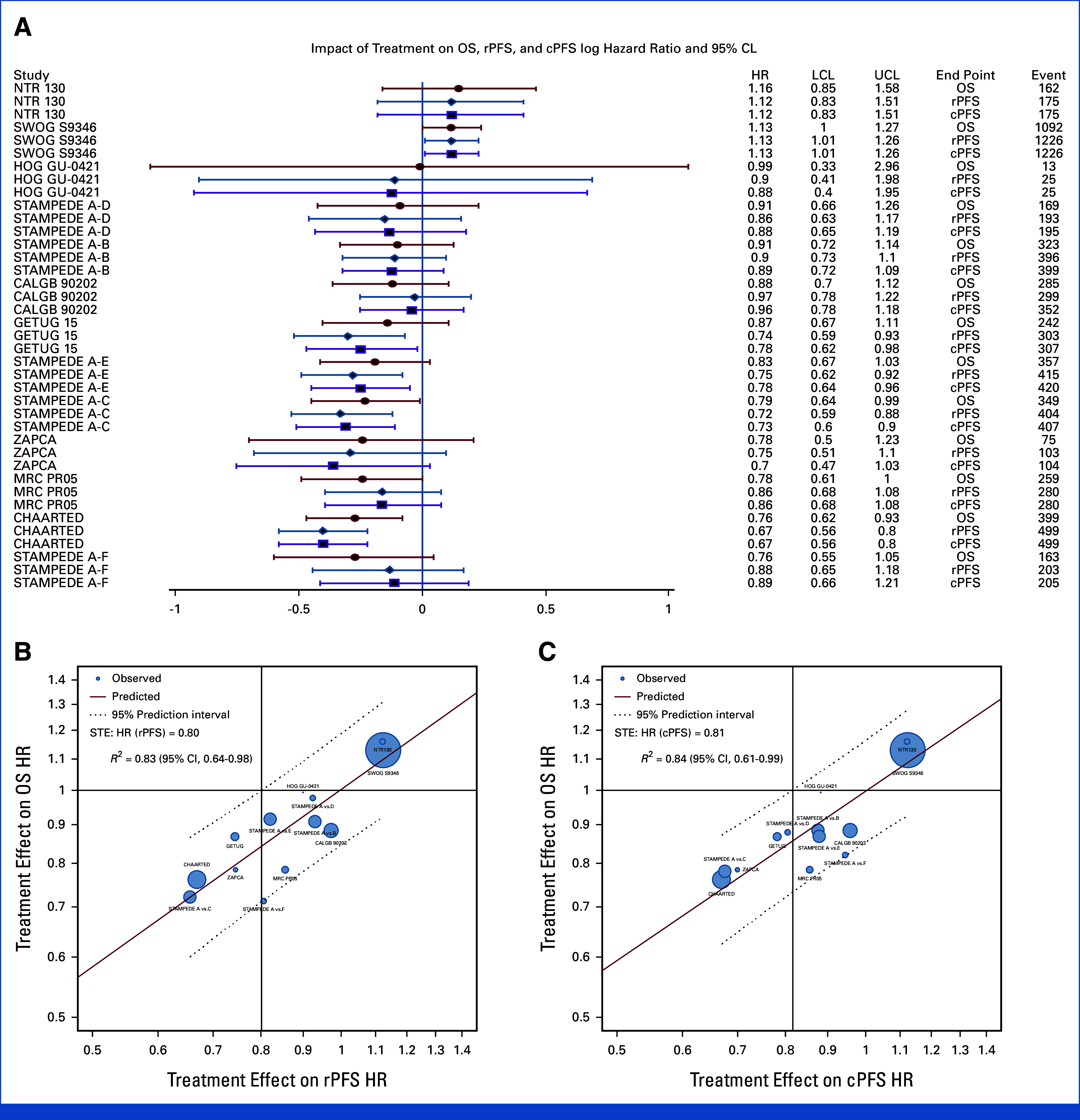
Treatment effects (HR) on OS, rPFS, and cPFS. (A) Forest plot of HRs of study-specific treatment effects (HR) on OS, rPFS, and cPFS. Trials are ordered by decreasing order of HR of OS. (B) OS HR versus cPFS. (C) OS HR versus rPFS. HRs were estimated from the PH model for each study. Circle size was the sample size of each trial, and regression was weighed by the inverse variance of log(HR) estimates for OS. cPFS, clinical progression-free survival; HR, hazard ratio; OS, overall survival; PH, proportional hazards; rPFS, radiographic progression-free survival; STE, surrogate threshold effect.

We present the study-specific treatment effects (HR) on OS, rPFS, and cPFS from the PH models in a forest plot (Fig 3A). *R*^2^ were 0.83 (95% CI, 0.64 to 0.98) and 0.84 (95% CI, 0.61 to 0.99) for log(HR)-OS versus log(HR)-rPFS and log(HR)-OS versus log(HR)-cPFS (Figs 3B and 3C), respectively. The STEs on OS for HR (rPFS) and for HR(cPFS) were 0.80 and 0.81, respectively, suggesting that risk reductions of 20% and 19% would predict a nonzero effect on OS (Figs 3A and 3B). The median *R*^2^ from the LOOCV was 0.78 for both ICEs (Data Supplement, Figs S3A and S3B), and the HRs fell within the 95% prediction intervals in all the 12 of 13 units indicating that the models were robust (Data Supplement, Figs S4A and S4B).

### Subgroup Analysis

#### Docetaxel Trials

**FIG 4.**
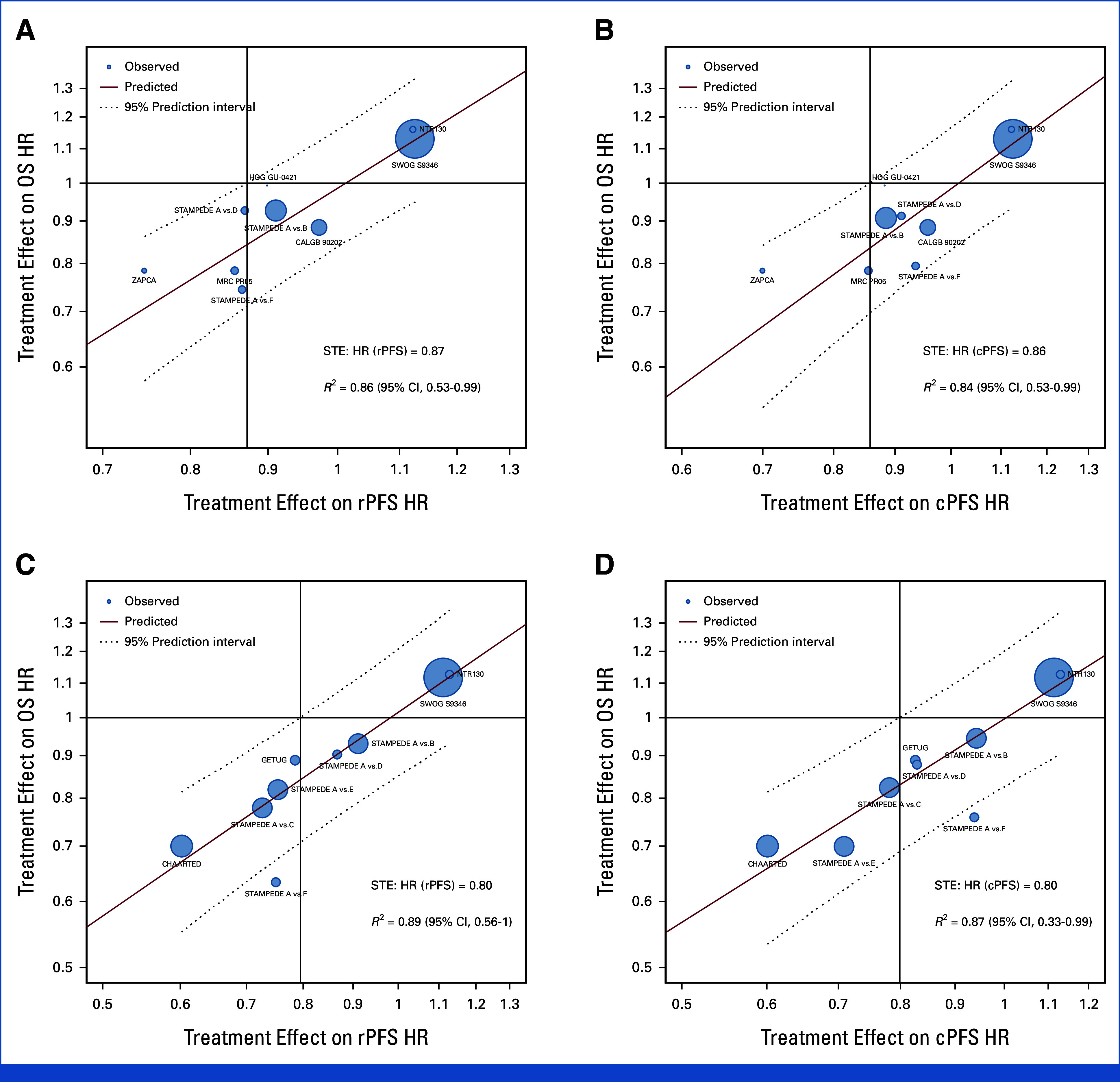
Treatment effects (HR) on OS versus treatment effects on ICEs: (A) OS HR versus rPFS HR for ADT; (B) OS HR versus cPFS HR for ADT; (C) OS HR versus rPFS for synchronous patients with sufficient follow-up; (D) OS HR versus cPFS for synchronous patients with sufficient follow-up. HRs were estimated from the PH model for each study. Circle size was the sample size of each trial, and regression was weighed by the inverse variance of log(HR) estimates for OS. cPFS, clinical progression-free survival; HR, hazard ratio; ICEs, intermediate clinical end points; OS, overall survival; PH, proportional hazards; rPFS, radiographic progression-free survival; STE, surrogate threshold effect.

Kendall's tau between OS and rPFS was 0.82 (95% CI, 0.81 to 0.83) and 0.71 (95% CI, 0.70 to 0.73) for ADT trials and ADT plus docetaxel (Data Supplement, Table S2A), respectively. The *R*^2^ from the WLR between 5-year OS and 3-year rPFS rates was 0.77 (95% CI, 0.40 to 0.96) and 0.49 (95% CI, 0.13 to 0.98) for the ADT trials alone and ADT plus docetaxel for patients with sufficient follow-up (Data Supplement, Table S2B), respectively. Similar associations were observed between 5-year OS and 3-year cPFS rates (Data Supplement, Tables S2A and S2B). For condition 2, *R*^2^ from WLR of log(HR)-OS versus log(HR)-rPFS were 0.86 (95% CI, 0.53 to 0.99) and 0.73 (95% CI, 0 to 1) for the ADT trials alone and ADT plus docetaxel (Data Supplement, Table S3), respectively. Similar associations were observed between OS and cPFS (Data Supplement, Tables S2A, S2B, and S3). The STEs on OS for HR(rPFS) and HR(cPFS) for patients enrolled on ADT trials were 0.87 and 0.86, respectively (Data Supplement, Table S4; Figs 4A and 4B). Conversely, the STEs on OS for HR(rPFS) and HR(cPFS) for patients enrolled on ADT plus docetaxel trials were not estimable (Data Supplement, Table S4).

#### Volume of Disease

Kendall's tau between OS and rPFS was 0.79 (95% CI, 0.78 to 0.81) and 0.71 (95% CI, 0.69 to 0.73) for patients with high-volume and low-volume disease, respectively (Data Supplement, Table S2A). The *R*^2^ from WLR between 5-year OS and 3-year rPFS rates were 0.77 (95% CI, 0.35 to 0.97) and 0.85 (95% CI, 0.59 to 0.98), respectively, for all patients with high-volume disease and those with sufficient follow-up (Data Supplement, Table S2B). By contrast, *R*^2^ between 5-year OS and 3-year rPFS rates was 0.43 (95% CI, 0.04 to 0.98) for patients with low-volume disease (Data Supplement, Table S2B). For condition 2, *R*^2^ from the WLR of log(HR)-OS versus log(HR)-rPFS were 0.87 (95% CI, 0.06 to 1.00) and 0.85 (95% CI, 0.16 to 1.00) for patients with high-volume and low-volume disease (Data Supplement, Table S3). Similar associations were observed between OS and cPFS (Data Supplement, Tables S2A, S2B, and S3). The STEs on OS for HR(rPFS) and HR(cPFS) for patients with high-volume disease were 0.71 and 0.69 (Data Supplement, Table S4 and Figs S5A and S5B) and were 0.60 and 0.68 for patients with low volume disease, respectively (Data Supplement, Table S4 and Figs S5C and S5D).

#### M1 Diagnosis Status

Kendall's tau between OS and rPFS and cPFS were 0.77 (95% CI, 0.76 to 0.78) and 0.78 (95% CI, 0.77 to 0.80) for synchronous patients (Data Supplement, Table S2A). At the trial level, *R*^2^ between the 5-year OS rates and 3-year rPFS rates was 0.62 (95% CI, 0.27 to 0.90) for synchronous patients with sufficient follow-up (Data Supplement, Table S2B). For condition 2, *R*^2^ from the WLR of log(HR)-OS versus log(HR)-rPFS were 0.82 (95% CI, 0.48 to 0.99) and 0.89 (95% CI, 0.56 to 1.00) for all synchronous patients and for those with sufficient follow-up (Data Supplement, Table S3). Similar associations were observed between OS and cPFS (Data Supplement, Tables S2A, S2B, and S3). The STE on OS for HR(rPFS) and HR(cPFS) for synchronous patients with mHSPC with sufficient follow-up was 0.80 (Data Supplement, Table S4; Figs 4C and 4D).

## DISCUSSION

In this surrogacy analysis, we observed a strong correlation between the ICEs and OS, with Kendall's tau above 0.84. Moreover, the *R*^2^ for both conditions 1 and 2 were high and met the a priori criteria. The STEs for rPFS and cPFS were 0.80 and 0.81, respectively. In this combined data set, there were a total of 3,861 deaths, of which 71% (2,735) were due to prostate cancer. This percent is low and could be due to missing information on cause of death in these older trials. The median OS among surviving patients was 6.1 years (range, 0.0-17.7). rPFS, a composite end point of progression or death, was experienced first by the majority of patients.

Men with mHSPC have heterogeneous outcomes. Because of this observation, we performed sensitivity analyses in patients with synchronous disease and high-volume disease and patients treated with ADT alone. We observed more striking results in overall and subgroup analysis when limiting the analysis to trials with sufficient follow-up. The STEs for rPFS in trials of ADT alone and in synchronous patients were 0.87 and 0.79, respectively. The lower STEs for docetaxel cohorts may be related to the smaller sample size, patient mix being fit for chemotherapy in the former and more indolent disease, and potential impact of salvage therapies for patients with metachronous disease.

A major challenge in designing trials in men with mHSPC is the choice of the primary end point. The observed median OS in our analysis is 4.3 years, whereas the median rPFS and cPFS are 2.4 and 2.3 years, respectively. The median OS outcomes are notably longer with docetaxel and the new androgen receptor (AR) inhibitor agents added to ADT, and this will have a major impact on the duration of future trials that use OS as a primary end point. Despite the long follow-up period (>5 years) in several of the recent trials, the median OS was not reached.^10,21-25^

Reflecting on the above, rPFS can be justified as the primary end point and would provide potential savings in costs and study duration if it were selected as the primary end point. The above trials had enrolled more than 1,100 patients and reported HRs for rPFS in the range of 0.39-0.54.^10,21-25^ Assuming a therapy with a treatment effect resulting in a HR(rPFS) of 0.5 or more, the trial duration can be shortened anywhere from 7 to 24 months (Data Supplement, Fig S6).

Although the results were slightly higher for cPFS, our preference is to use rPFS as it is a more objective end point than cPFS. Reporting of symptoms and reasons for treatment switch is viewed as subjective by regulatory agencies and is often plagued by missing data. Notably, rPFS in this analysis was ascertained by investigator assessment and did not require repeat scan confirmation with new lesions and other PCWG3 criteria. Our results agree with another meta-analysis on the basis of ADT trial-level data.^26^

We advocate for the use of IPD because the analysis based on them will be statistically rigorous.^27^ By having access to the IPD, we were able to harmonize the definition of the ICEs, confirm reported results, implement surrogacy analysis at the individual and trial levels, perform sensitivity analyses with different censoring assumptions on the ICEs, and conduct subgroup analysis and avoid potential bias.^28,29^

There are several strengths to this pooled analysis. First, we included IPD from more than 6,300 patients enrolled in randomized phase II and phase III trials. In validating the ICEs, we tested surrogacy conditions at both the individual and trial levels, and there was sufficient statistical power to identify surrogate end points of OS. Second, by identifying surrogate outcomes for OS, we will accelerate the evaluation of novel treatments in future trials. Third, we were able to perform uniform and subgroup analyses across different trials which cannot be performed without pooling the IPD. Finally, we followed the PRISMA and ReSEEM guidelines in performing and reporting the results.^30^

There are a few potential limitations in this ICE analysis. First, the patient population consists of men with mHSPC who met the inclusion criteria for the clinical trials and may not represent current patients with mHSPC. Second, we noted a smaller *R*^2^ and STE when the analysis was restricted to trials of docetaxel plus ADT. This might be due to the potential postprogression therapy which would have a major effect on OS and may have affected the identification of ICEs in patients treated with docetaxel plus ADT.^31^ Finally, we recognize the limitation in extrapolation of ICEs to settings where the biologic mode of action may be different from the ones used to validate the surrogates. In STAMPEDE, different relationships between rPFS and OS were observed in patients treated with docetaxel and abiraterone. This could be due to prostate-specific antigen control which is much longer with potent AR inhibition (TS plus more AR inhibition [abiraterone, enzalutamide, darolutamide, apalutamide]), and the longer time to progression is expected with chronic potent AR inhibition versus weak ADT (TS alone and TS plus docetaxel).^10,32^ The modest impact on OS from the current therapeutic options for progression after chronic potent AR inhibition is likely to result in maintaining a strong surrogacy of rPFS and cPFS with OS. However, the current findings are limited to studies with TS as the backbone therapy. Validation of these ICEs in trials with drugs having other mechanisms of action such as ADT plus the potent AR inhibitors is required and planned.

In summary, both rPFS and cPFS appear to be promising surrogate end points for OS when the backbone of therapy is ADT alone. The STE of 0.80 or higher makes it viable for either rPFS or cPFS to be used as the primary end point as a surrogate for OS in phase III mHSPC trials and would expedite trial conduct. Validation of these ICEs in trials with drugs having other mechanisms of action such as ADT plus the potent AR inhibitors is planned.

## Data Availability

The data from the industry trials were obtained from STOPCAP M1 repository stored at the Medical Research Council Clinical Trials Unit at University College London. Data from the National Cancer Institute (NCI) NCTN Trials (CALGB 90202, E3805, and SWOG 9346) were obtained from the NCI NCTN/NCORP Data Archive or SWOG. Requests for access to the study data can be submitted through the Medical Research Council, the NCI NCTN/NCORP Data Archive, and directly from the sponsors.
