# Supplementary material for: Radiographic Progression-Free Survival and Clinical Progression-Free Survival as Potential Surrogates for Overall Survival in Men With Metastatic Hormone-Sensitive Prostate Cancer
Source: J Clin Oncol. Author manuscript; Available in PMC 2024 Oct 2. (PMC10950170; doi:10.1200/JCO.23.01535)
Supplement: Supplementary Data [file EMS198672-supplement-Supplementary_Data.pdf]

Supplementary Data for the Article: Radiographic Progression-Free Survival and Clinical Progression-Free Survival as Potential Surrogates for Overall Survival in Men With Metastatic Hormone-Sensitive Prostate. Halabi, S et al.

Table S1. Clinical Trials in mHSPC Included in The Analysis. Trials are ordered by the date of beginning of trial enrollment.

Trial (NCTID)	Years of recruitment	Sample Size	Median Follow-up (years, range)
**MRC PR05 (ISRCTN38477744)**<br>Oral sodium clodronate vs Placebo	June 1994-July 1998	311	6.6 (3.0-9.7)
**SWOG S9346 (NCT00002651)**<br>Continuous androgen deprivation therapy (ADT) vs Intermittent ADT	May 1995-Sep 2008	1535	9.8 (0.0-17.7)
**NTR130 (NL99)**<br>Continuous ADT vs. Intermittent ADT	January 2000 – July 2013	258	6.1 (0.0-11.6)
**HOG GU-0421 (NCT00216060)**<br>Residronate vs. Placebo	December 2003-August 2005	63	3.4 (0.0-4.7)
**CALGB 90202 (NCT00079001)**<br>Zoledronic acid (ZA) vs. ADT	January 2004-May 2012	645	3.2 (0.0-8.0)
**GETUG 15 (NCT00104715)**<br>ADT + docetaxel vs. ADT	October 2004-December 2008	385	7.0 (0.0-9.5)
**STAMPEDE (NCT00268476)**<br>•SOC + ZA (A vs. B)<br>•SOC + docetaxel (A vs. C)<br>•SOC + celecoxib (A vs. D)<br>•SOC + ZA + docetaxel (A vs .E)<br>•SOC + ZA + celecoxib (A v. F)	October 2005-March 2013<br>October 2005-March 2013<br>October 2005-April 2011<br>October 2005-March 2013<br>October 2005-April 2011	1090<br>1086<br>565<br>1090<br>567	5.0 (0-10.1)<br>6.5 (0-12.0)<br>6.8 (0-10.0)<br>6.4 (0-11.4)<br>6.8 (0-10.0)
**CHAARTED (NCT00309985)**<br>ADT + docetaxel vs. ADT	July 2006- December 2012	790	4.5 (0.0-8.8)
**ZAPCA (NCT00685646)**<br>ZA+ combined ADT vs. combined ADT	May 2008-December 2010	227	3.9 (0.1-5.9)

Table S2A. Results at the Patients Level for Condition 1 in the Overall and the Subgroup of Patients

		Correlation between rPFS and OS	Correlation between cPFS and OS
	No of patients (No of units)	*Kendall's Tau (95%CI)*	*Kendall's Tau (95%CI)*
All patients	6390 (13)	0.83 (0.82-0.84)	0.85 (0.85-0.86)
Patients with sufficient follow-up*	5475 (10)	0.84 (0.83-0.84)	0.86 (0.85-0.87)
**Type of therapy**			
ADT+docetaxel	2627 (04)	0.71 (0.70-0.73)	0.72 (0.71-0.74)
ADT	4487 (09)	0.82 (0.81-0.83)	0.83 (0.82-0.84)
ADT (Patients with sufficient follow-up)	3572 (06)	0.85 (0.84-0.85)	0.86 (0.85-0.87)
**Volume of Disease ****			
High volume	2294 (7)	0.79 (0.78-0.81)	0.77 (0.76-0.78)
High volume (patients with sufficient follow-up*)	2107 (5)	0.78 (0.76-0.80)	0.76 (0.74-0.78)
Low volume	1787 (6)	0.71 (0.69-0.73)	0.70 (0.68-0.72)
Low volume (Patients with sufficient follow-up*)	1697(5)	0.74 (0.73-0.76)	0.72 (0.70-0.74)
**M1 Diagnosis Status**			
Synchronous (de novo)	4891 (12)	0.77 (0.76-0.78)	0.78 (0.77-0.80)
Synchronous (Patients with sufficient follow-up*)	4184 (09)	0.78 (0.77-0.79)	0.78 (0.77-0.80)

*Excluding CALGB 90202, HOG GU-01, ZAPCA as their median follow-up time is less than 4 years.
** Three STAMPEDE Comparisons A vs B, A vs D, A vs F were excluded in the disease volume subgroup as the data was not collected
OS: Overall survival, rPFS: Radiographic progression free survival, cPFS: Clinical progression free survival, ADT: Androgen deprivation therapy

Table S2B. Results at the Trial Level for Condition 1 for Overall and Subgroup of Patients

		**Correlation between 3-year rPFS and 5-year OS (by trial and arm)**	**Correlation between 3-year cPFS and 5-year OS (by trial and arm)**
	No of patients (No of units)	*Weighted by OS* *R^2 (95% CI)*	*Weighted by OS* *R^2 (95% CI)*
All patients	6390 (13)	0.62 (0.29-0.89)	0.74 (0.49-0.90)
Patients with sufficient follow-up*	5475 (10)	0.74 (0.40-0.96)	0.73 (0.43-0.94)
**Type of therapy**			
ADT+Docetaxel	2627 (04)	0.49 (0.13-0.98)	0.50 (0.05-0.99)
ADT Alone	4487 (09)	0.77 (0.40-0.96)	0.89 (0.73-0.98)
ADT (Patients with sufficient follow-up*)	3572 (06)	0.91 (0.69-0.99)	0.89 (0.63-0.99)
**Volume of Disease****			
High volume	2294 (7)	0.77 (0.35-0.97)	0.79 (0.47-0.97)
High volume (Patients with sufficient follow-up*)	2107 (5)	0.85 (0.59-0.98)	0.85 (0.59-0.98)
Low volume	1787 (6)	0.43 (0.04-0.98)	0.35 (0.01-0.97)
Low volume (Patients with sufficient follow-up*)	1697(5)	0.35 (0.02-0.99)	0.26 (0.01-0.98)
**M1 Diagnosis Status**			
Synchronous (de novo)	4891 (12)	0.57 (0.28-0.84)	0.67 (0.40-0.92)
Synchronous (Patients with sufficient follow-up*)	4184 (09)	0.62 (0.27-0.90)	0.61 (0.30-0.91)

*Excluding CALGB 90202, HOG, ZAPCA as their median follow-up time is less than 4 years.
** Three STAMPEDE Comparisons A vs B, A vs D, A vs F were excluded in the disease volume subgroup as the data was not collected. OS: Overall survival, rPFS: Radiographic progression free survival, cPFS: Clinical progression free survival, ADT: Androgen deprivation therapy

Table S3. Results at the Trial Level for Condition 2 for Overall and Subgroup of Patients

		Correlation between Log-HR (OS) vs Log-HR (rPFS) (by trial)	Correlation between Log-HR (OS) vs Log-HR (cPFS) (by trial)
	No of patients (No of units)	Weighted by OS R^2 (95% CI)	Weighted by OS R^2 (95% CI)
All patients	6390 (13)	0.83 (0.64-0.98)	0.84 (0.61-0.99)
Patients with sufficient follow-up*	5475 (10)	0.86 (0.54-0.99)	0.86 (0.58-0.99)
**Type of therapy**			
ADT+Docetaxel	2627 (4)	0.73 (0-1.0)	0.88 (0-1.00)
ADT Alone	4487 (9)	0.86 (0.53-0.99)	0.84 (0.53-0.99)
ADT (Patients with sufficient follow-up*)	3572 (6)	0.89 (0.22-0.99)	0.89 (0.12-0.99)
**Volume of Disease ****			
High volume	2294 (7)	0.87 (0.06-1.00)	0.84 (0.04-0.99)
High volume (Patients with sufficient follow-up*)	2107 (5)	0.93 (0-1.00)	0.94 (0-1.00)
Low volume	1787 (6)	0.85 (0.16-1.00)	0.90 (0.36-1.00)
Low volume (Patients with sufficient follow-up*)	1697(5)	0.89 (0.32-1.00)	0.91 (0-1.00)
**M1 Diagnosis Status**			
Synchronous (de novo)	4891 (12)	0.82 (0.48-0.99)	0.81 (0.44-0.99)
Synchronous (Patients with sufficient follow-up*)	4184 (9)	0.89 (0.56-1.00)	0.87 (0.33-0.99)

*Excluding CALGB 90202, HOG, ZAPCA as their median follow-up time is less than 4 years.
** Three STAMPEDE Comparisons A vs B, A vs D, A vs F were excluded in the disease volume subgroup as the data was not collected. OS: Overall survival, rPFS: Radiographic progression free survival, cPFS: Clinical progression free survival, ADT: Androgen deprivation therapy

Table S4. Surrogacy Threshold Effect (STE) in all Patients and Subgroups of Patients

	No of patients (No. of units)	STE for rPFS	STE for cPFS
All patients	6390 (13)	0.80	0.81
Patients with sufficient follow-up*	5475 (10)	0.79	0.80
**Type of Therapy**	6390 (13)	0.80	0.81
ADT+Docetaxel	2627 (4)	NE	NE
ADT Alone	5662 (9)	0.87	0.86
ADT (Patients with sufficient follow-up*)	3572 (6)	0.84	0.84
**Volume of Disease**			
High volume	2294 (7)	0.71	0.69
High volume (Patients with sufficient follow-up*)	2107 (5)	0.70	0.73
Low volume	1787 (6)	0.60	0.68
Low volume (Patients with sufficient follow-up*)	1697(5)	0.55	0.60
**M1 Diagnosis Status**			
Synchronous (de novo)	4891 (12)	0.79	0.80
Synchronous (Patients with sufficient follow-up*)	4184 (9)	0.80	0.80

*Excluding CALGB 90202, HOG, ZAPCA as their median follow-up time is less than 4 years.
**Three STAMPEDE Comparisons A vs B, A vs D, A vs F were excluded in the disease volume subgroup as the data was not collected.
OS: Overall survival, rPFS: Radiographic progression free survival, cPFS: Clinical progression free survival, ADT: Androgen deprivation therapy, NE=not estimable

Figure S1. PRISMA Flowchart of Section and Participation of Randomized Clinical Trials in mHSPC Men

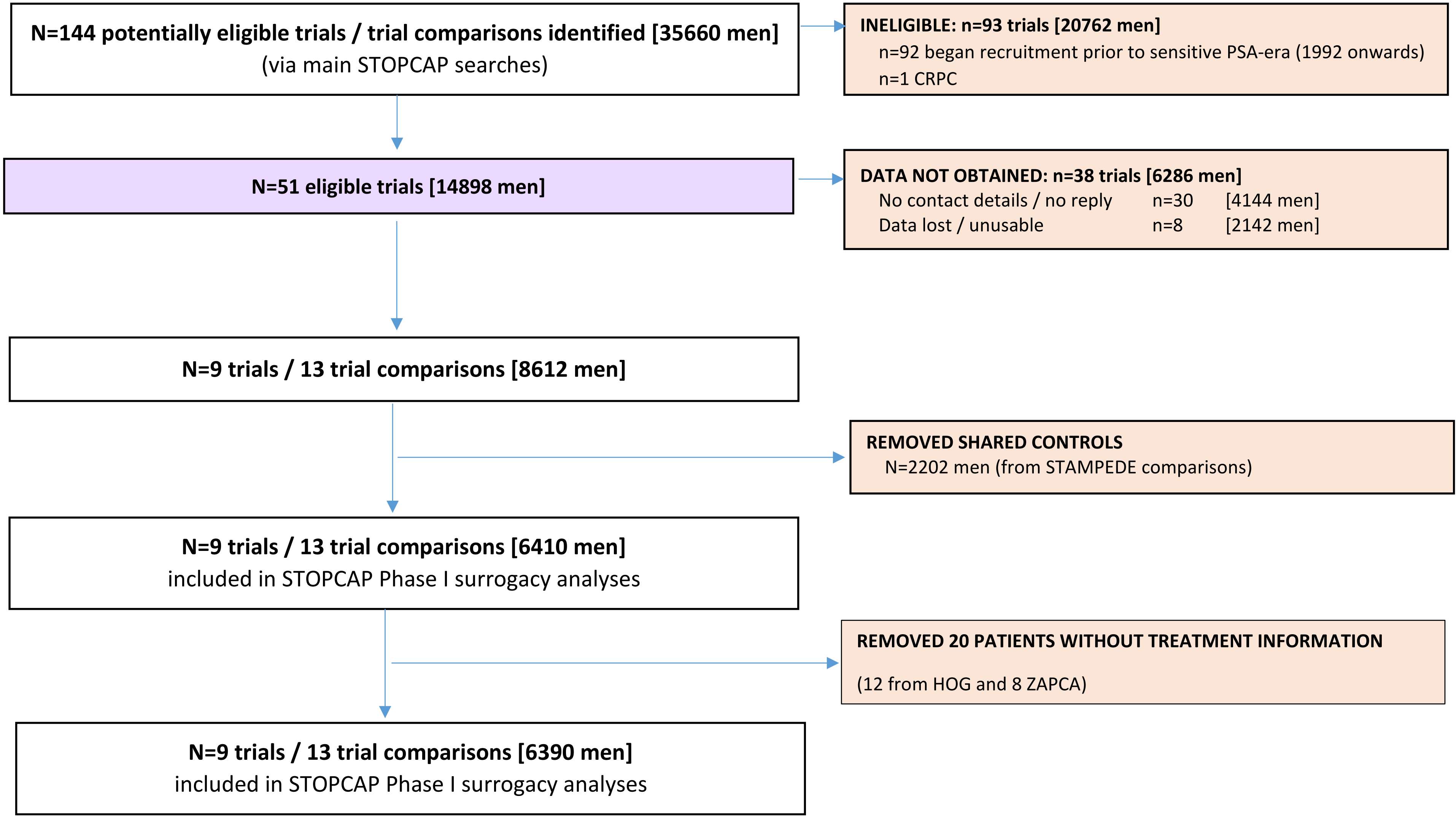

Figures S2A-S2B. Estimated hazard functions for: (A) OS and rPFS and (B) OS and cPFS

A.

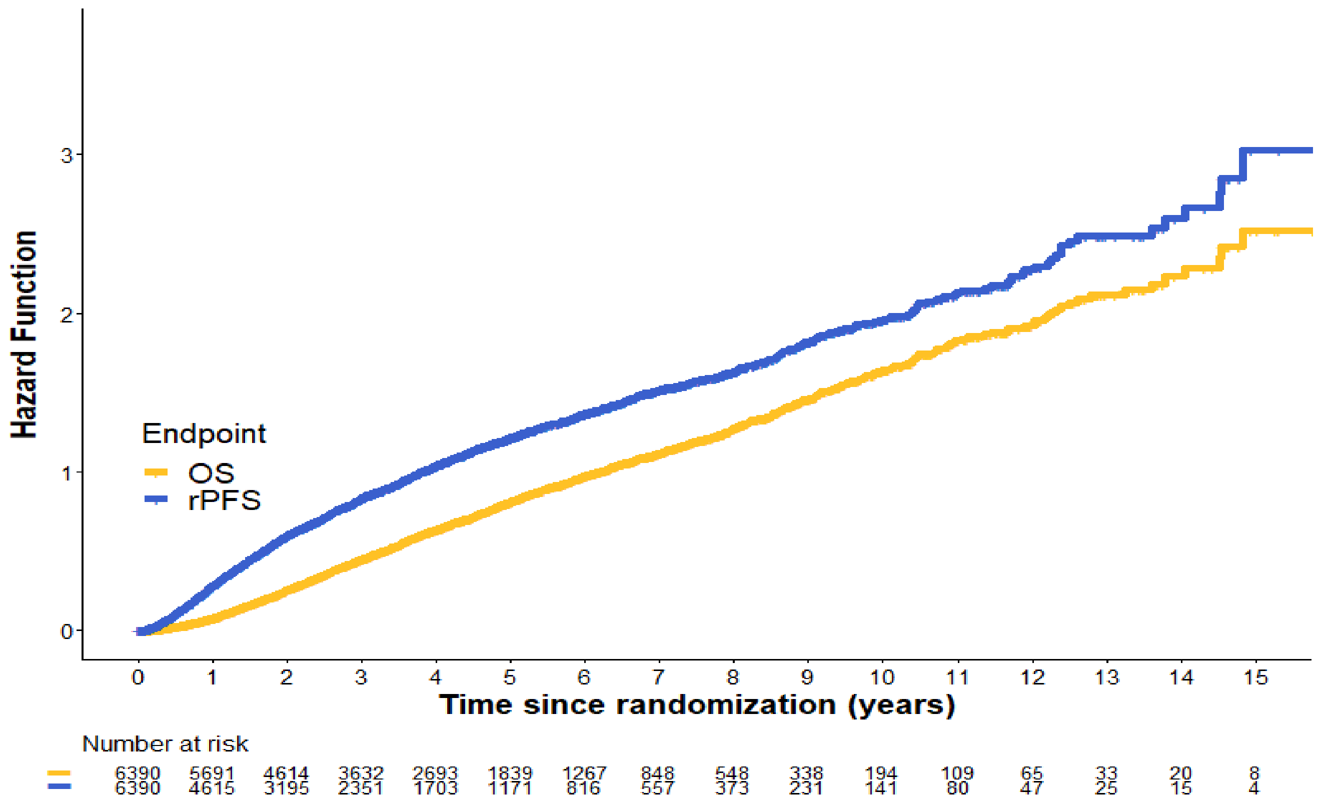

B.

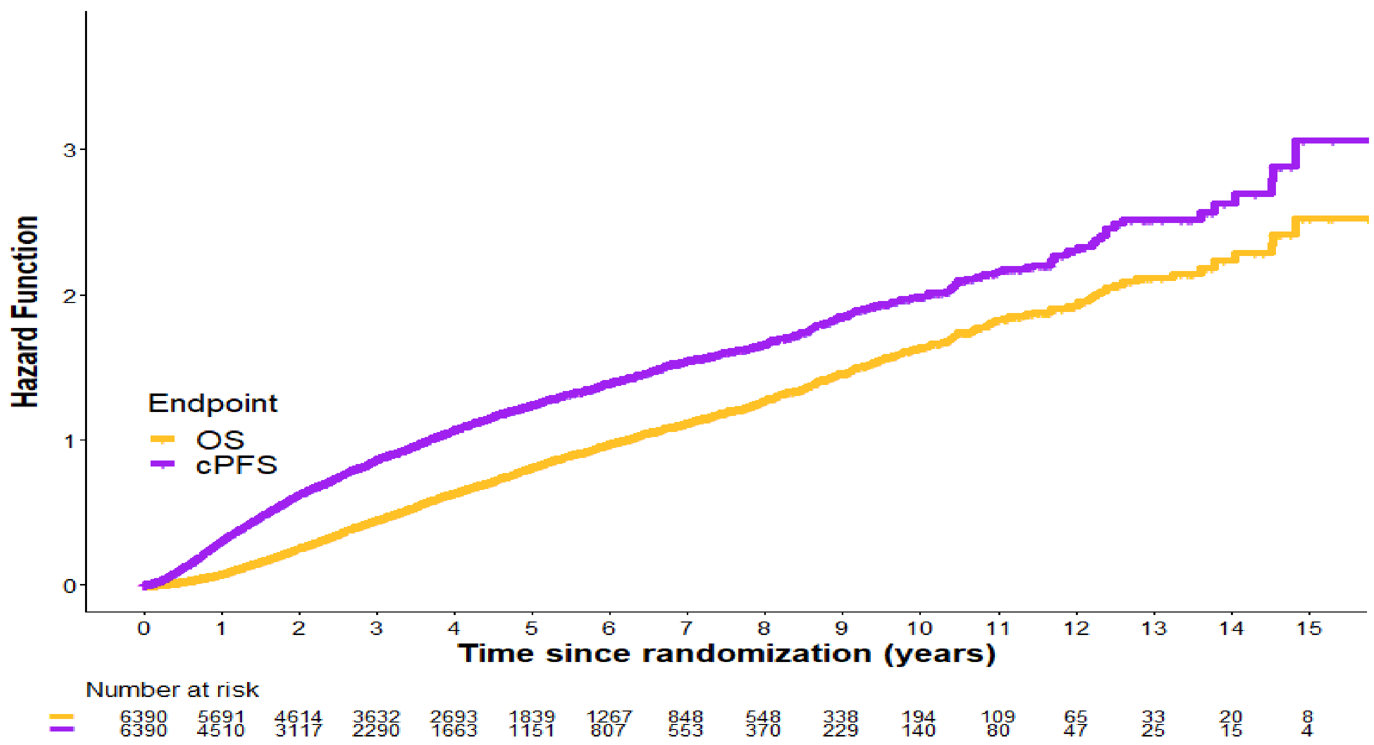
Hazard Function
0
1
2
3
Endpoint
OS
cPFS
0 1 2 3 4 5 6 7 8 9 10 11 12 13 14 15
Time since randomization (years)
Number at risk
6390 5691 4614 3632 2693 1839 1267 848 548 338 194 109 65 33 20 8
6390 4510 3117 2290 1663 1151 807 553 370 229 140 80 47 25 15 4

Figures S3A-3B. Leave-One-Out-Cross Validation (13 trials comparisons combined): R^2 between HR(OS) and (A) rPFS and (B) cPFS.

A. R-squared between Log-HR(OS) and log-HR(rPFS)
Median (range): 0.78 (0.61, 0.84)

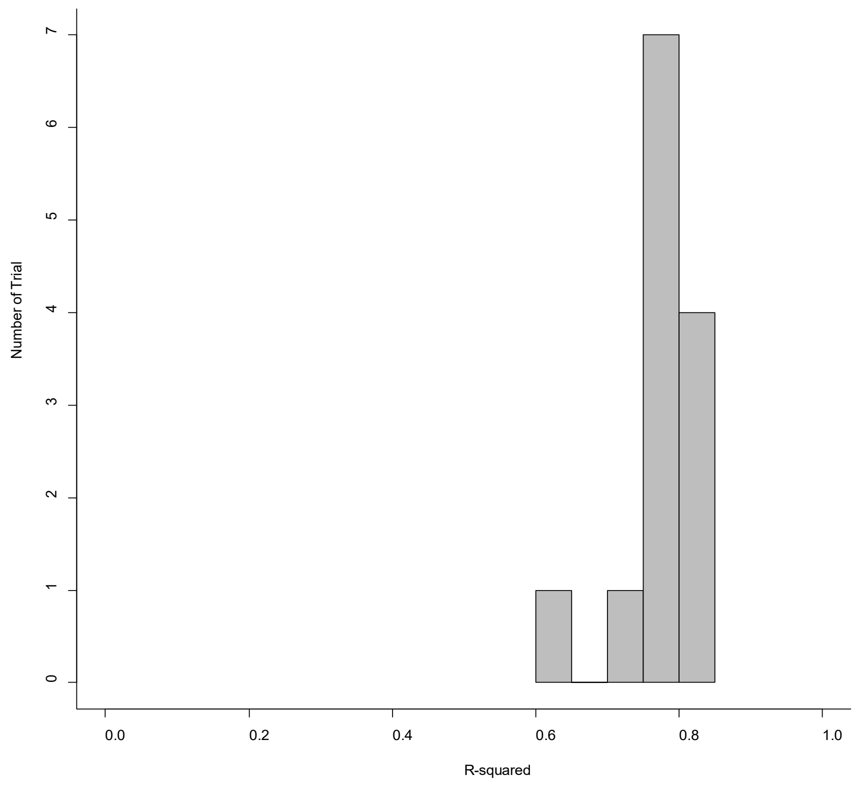

B. R-squared between Log-HR(OS) and log-HR(cPFS)
Median (range): 0.78 (0.62, 0.86)

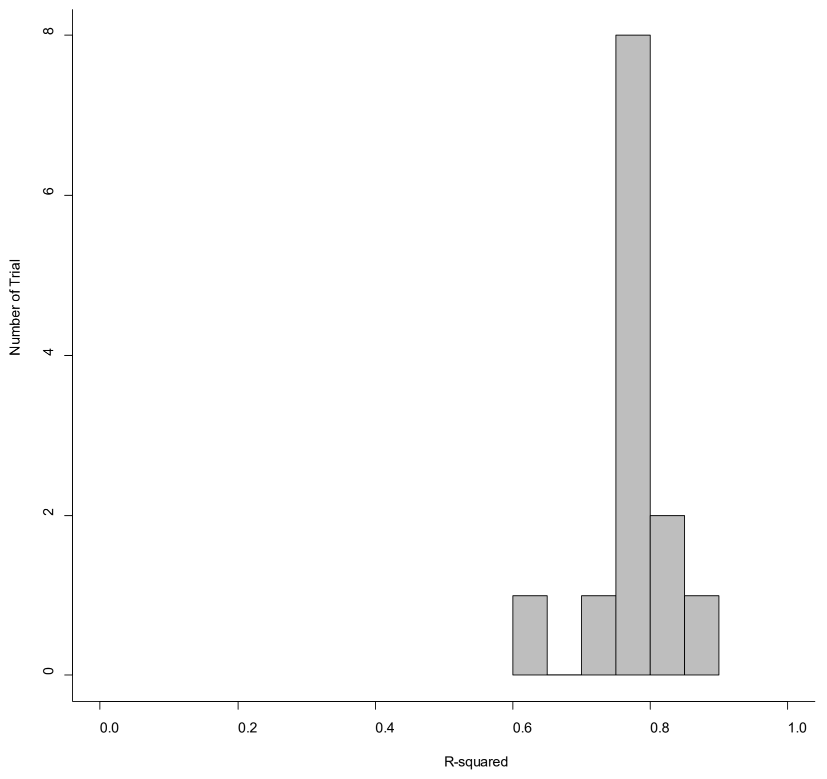

Figures S4A-S4B. Leave-one-out-cross-validation: observed versus predicted treatment effects on OS based on: (A) HR (rPFS) and (B) HR (cPFS). Each trial was left out once and the WLR analysis (i.e. treatment effect on OS versus treatment effect on the ICEs) was rebuilt on the remaining n-1 trials. The model was then applied to the left-out trial to obtain the predicted treatment effect (i.e., log (HR)) on OS or ICEs, along with 95% prediction intervals (accounted for the weight of the left-out trial). R^2 was also calculated from the remaining n-1 trials model to evaluate the impact of a single trial on the correlation between treatment effects on endpoints. Black squares correspond to predicted hazard ratios (HR) on OS using the observed HR on surrogates of that particular trial, based on the regression model built on all the other (i.e. the remaining n-1) trials; red circles correspond to the observed HR on OS of that particular trial; horizontal lines correspond to 95% prediction intervals.

(A) Prediction for HR(OS) based on HR (rPFS) [6,390]

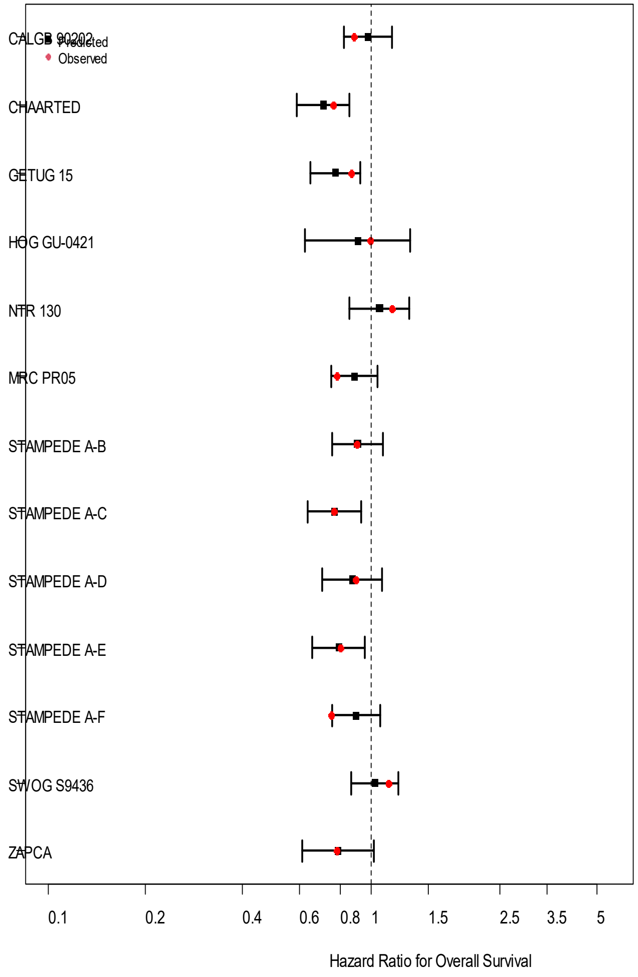

(B) Prediction for HR(OS) based on HR (cPFS) [n=6,390]

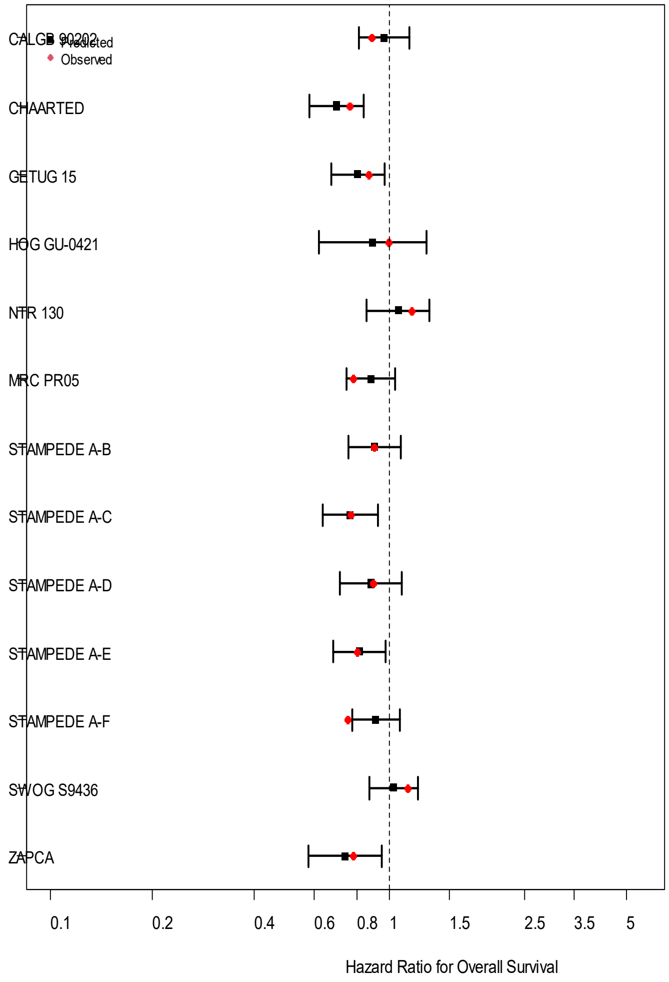

Figures S5A-5D. Treatment effects (hazard ratio [HR]) on overall survival (OS) versus treatment effects on ICEs: (A) OS HR versus radiographic progression-free survival (rPFS) HR for high volume disease; (B) OS HR versus cPFS HR for high volume disease; (C) OS HR versus rPFS HR for low volume disease; (D) OS HR versus cPFS for low volume disease. Circle size was the sample size of each trial and regression was weighed by the inverse variance of log(HR) estimates for OS. STE, surrogate threshold effect.

A. OS HR versus rPFS HR for high volume disease

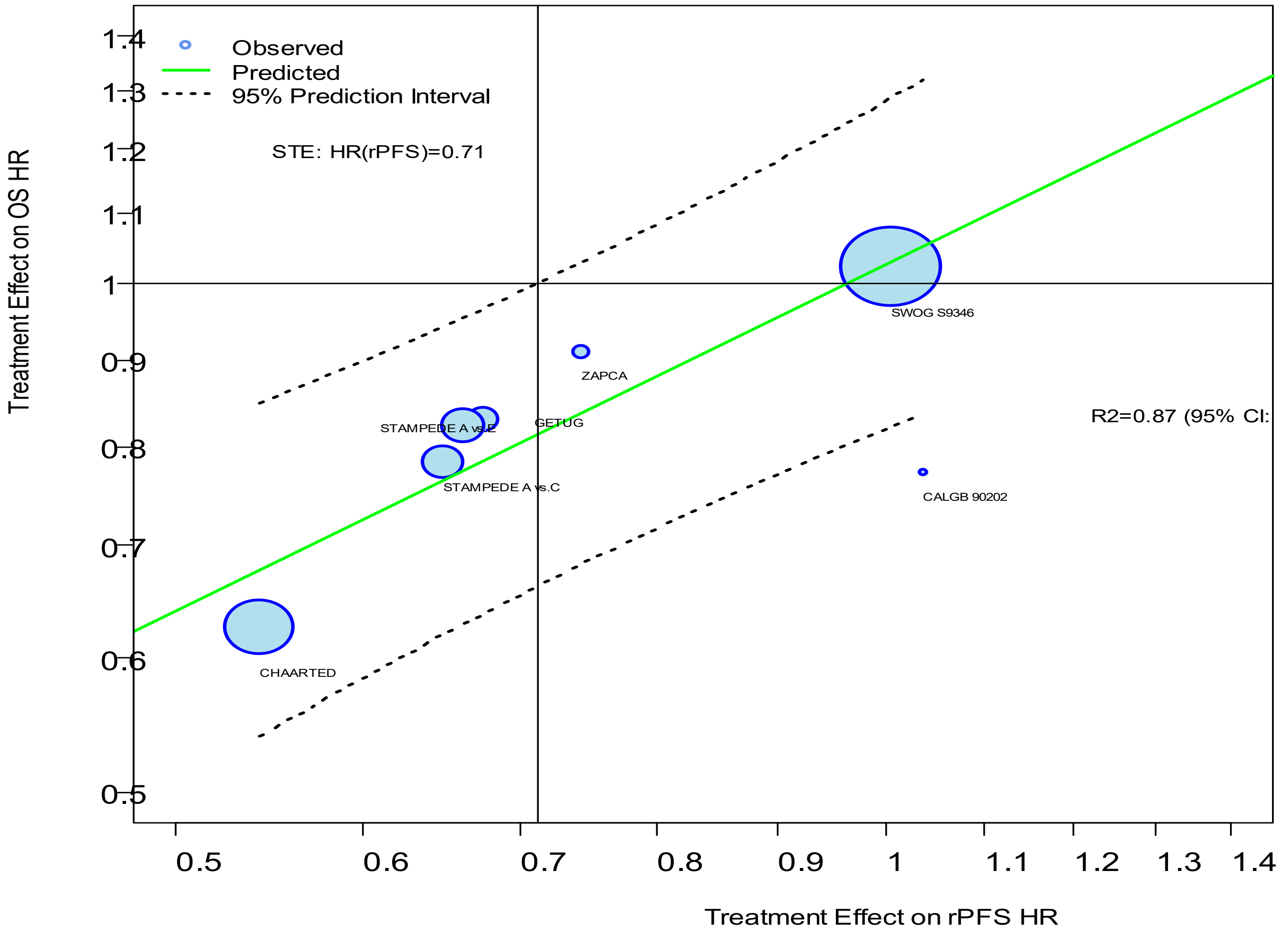

B. OS HR versus cPFS HR for high volume disease

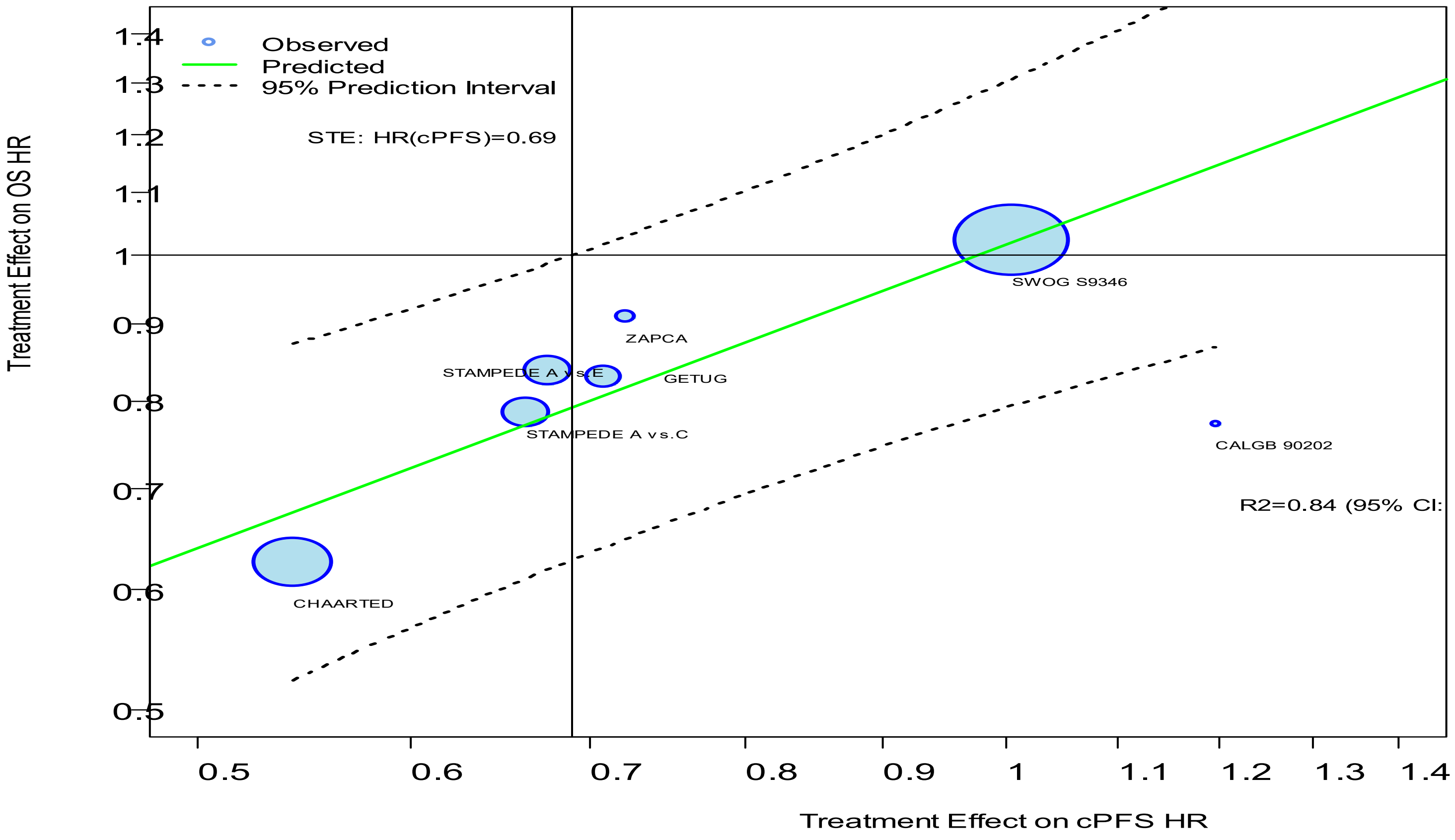

C. OS HR versus rPFS HR for low volume disease

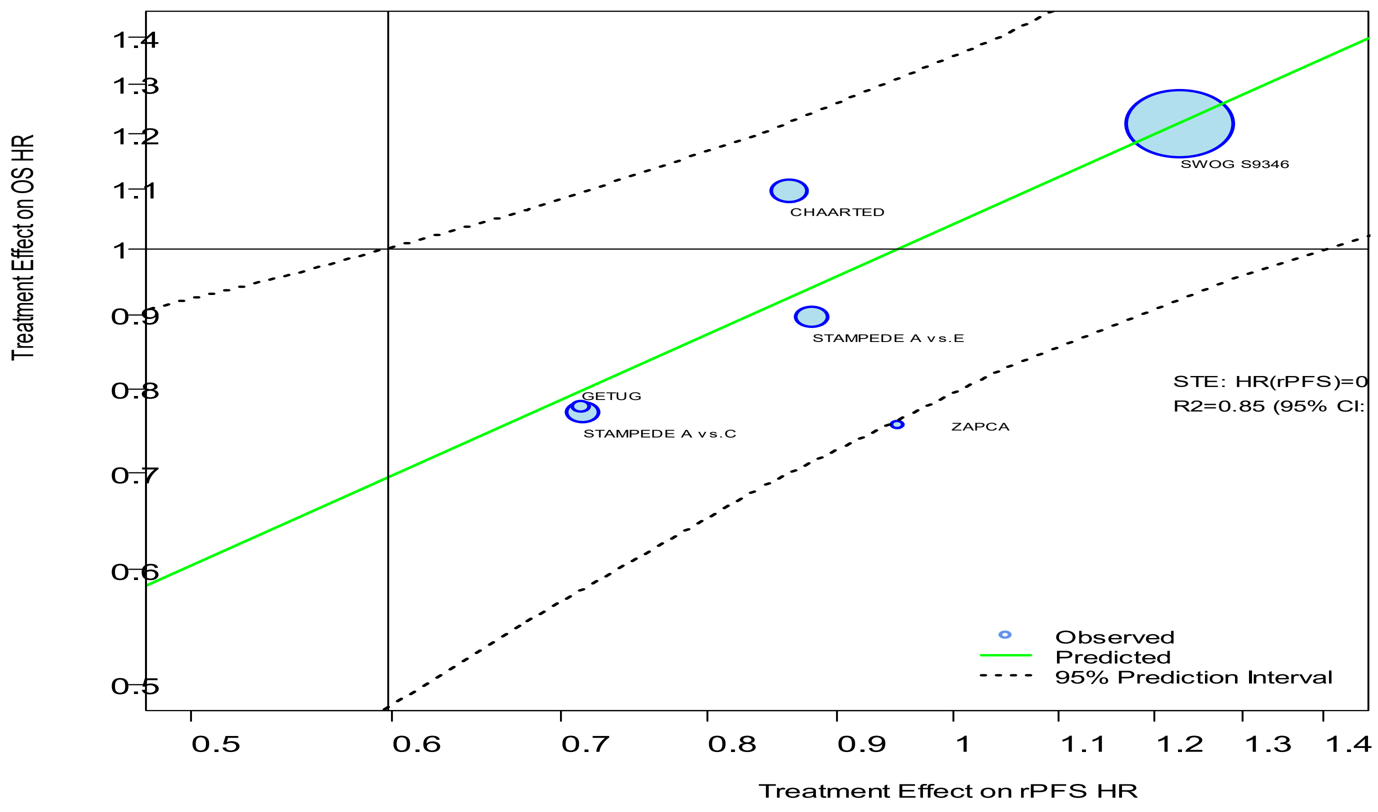

D. OS HR versus cPFS HR for low volume disease

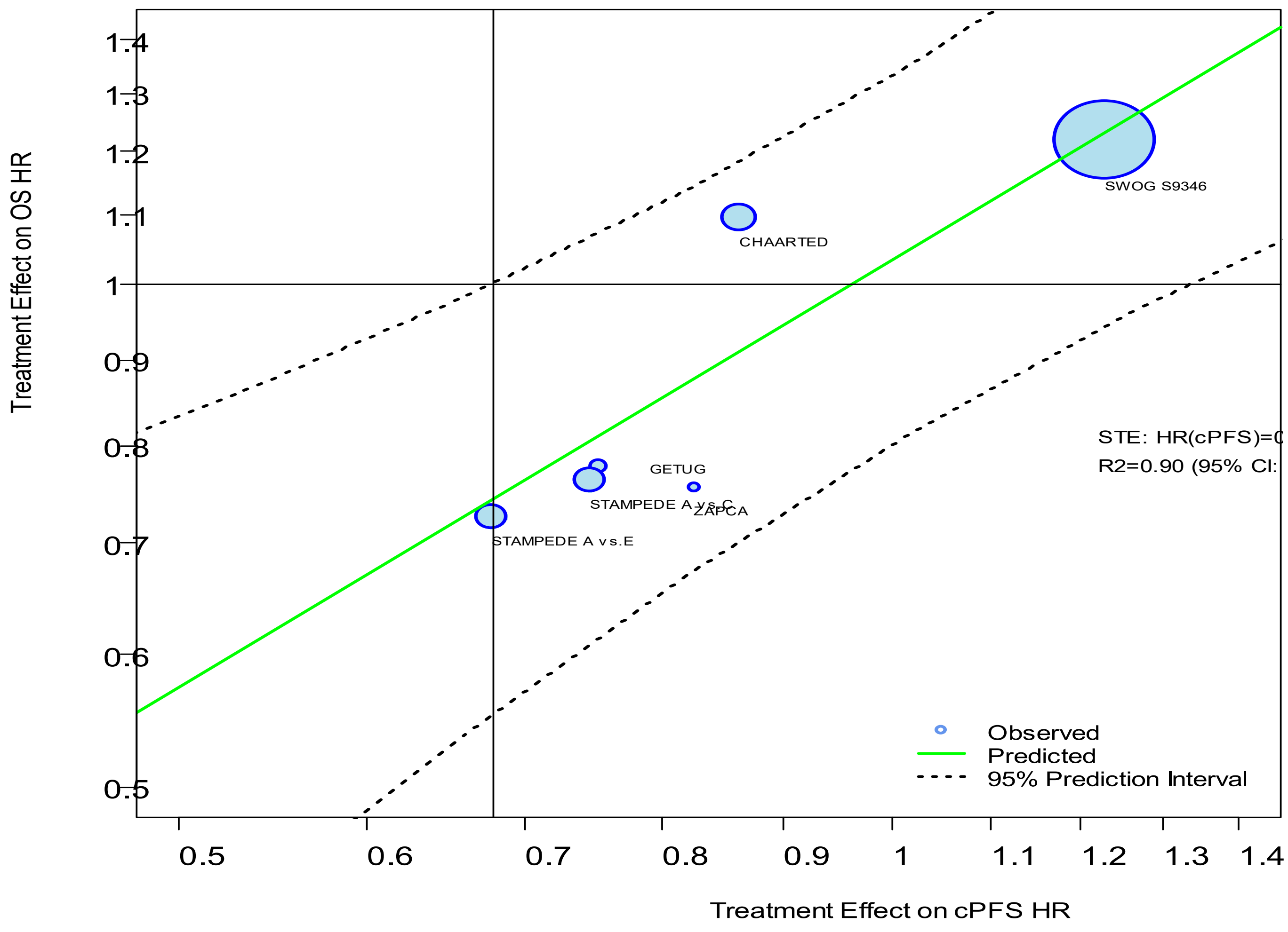

Figure S6. Total study duration (months) required in future trials using rPFS HRs and testing using the surrogate threshold effect (STE; solid lines) or using the predicted overall survival (OS) HRs from the weighted linear regression (dashed lines). rPFS would be the preferred primary end point for HR(OS) of <0.60, whereas OS would be the preferred primary end point for HR(OS) of ≥0.60 (vertical solid lines). Design assumptions include: 60-months rPFS rate of 15.6%, 60-months OS rate of 48.4% (0.0310 and 0.0121 hazard under the exponential distribution), accrual rate of 20 patients/month, one-sided type I error of 0.025 and type II error of 0.20.

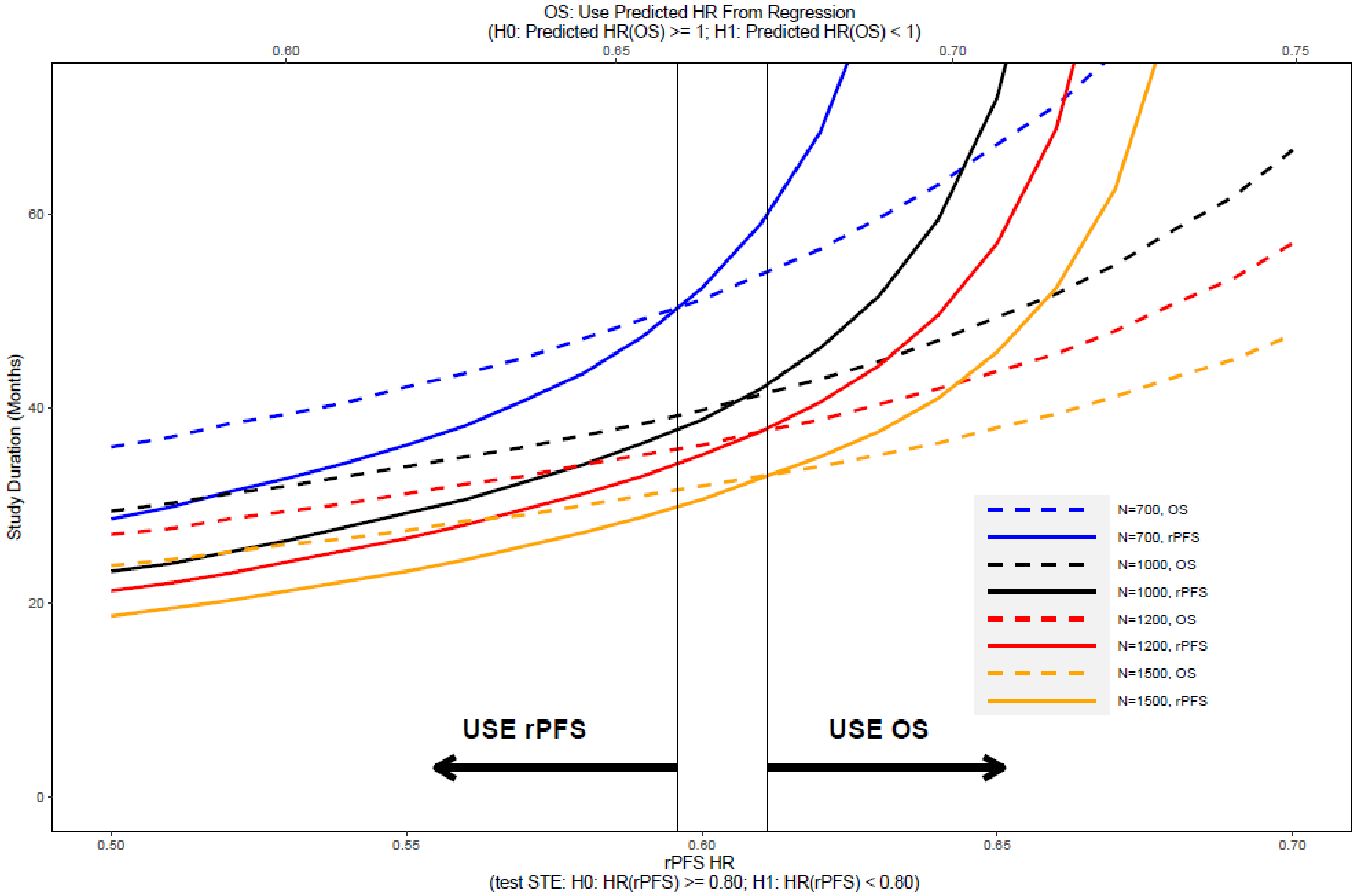

Description of the Two-Stage Meta-Analytic Approach

We investigated the intermediate clinical endpoints (ICEs) as potential surrogates for overall survival (OS) through the two-stage validation analysis to estimate the trial-specific treatment effects on these endpoints. We tested for conditions that need to be satisfied. The first condition was that the surrogate (rPFS, cPFS) and the true endpoints (OS) are strongly correlated (condition 1). The second condition was to test that the effect of the intervention on the surrogate was sufficiently correlated with the effect on the true endpoint (condition 2). Within this framework, the ICEs was assessed at both the trial and the individual patient levels for its ability to predict the effect of the treatment on OS.

<u>For condition 1:</u> At the individual patient level: we utilized different copulas (Clayton, Hougaard, Joe, and Plackett). Using the marginal distributions, we constructed bivariate copula models to estimate the association parameter and the correlation coefficient (Kendall τ) between the two time-to-event endpoints. The Clayton copula was chosen for both ICEs as it provided the best model fitness based on the regularized goodness of fit tests.

At the trial level: we performed weighted linear regression analysis and obtained trial and arm-specific Kaplan-Meier (KM) estimates of OS at 5-years vs. the Kaplan-Meier estimate of the ICEs at 3-years (rPFS, cPFS). Regression was weighted by the inverse variances of the OS.

<u>For condition 2</u>: We modelled the effect of the treatment on the marginal distribution of the ICEs and OS by employing the proportional hazards (PH) regression, where the treatment effects quantified as log hazard ratios was estimated with treatment arm as the only variable in the PH models. Each clinical trial was analyzed separately and we obtained trial-specific of the hazard ratio that estimated the treatment effect predicting the ICEs and OS (thus three models for each endpoint). We constructed a forest plot to present the effect of the treatment (HRs) on the ICEs and OS. Lastly, we computed the surrogate threshold effect (STE) which was defined as the

minimum treatment effect on the surrogate necessary to predict a non-zero effect on OS in a future trial. A large value of STE indicated the need to observe a large treatment effect on the surrogate endpoint in order to conclude a nonzero effect on OS.

S7 Splitting of the STAMPEDE Controls

STAMPEDE is a platform trial where the comparison arms share controls (standard of care (SOC) denoted as A). The concept behind splitting the controls was to generate pseudo independent trials by randomly splitting the common control arm between all treatment contrasts. The control arms were mutually exclusive between the pseudo-trials and thus the generated pseudo-trials were considered independent.

Random Splitting Approach

STAMPEDE comparisons A vs. D (celecoxib) and A vs. F (SOC+zoledronic acid+celecoxib) had the same date of randomization and trial length while A vs. B (SOC+ zoledronic acid), A vs. C (SOC+docetaxel) and A vs. E (SOC+zoledronic acid+docetaxel) had extended trial length. There were 377 common patients who were randomized into control group in all five STAMPEDE comparisons. Three hundred forty seven patients in the control arm were shared by comparisons A vs. B, A vs. C and A vs. E. The five independent datasets were created based on the following three steps:

1. We split the 377 controls in the first time frame (2005-2011) into five groups of nearly equal sizes and equal numbers of events with respect to both the OS and rPFS endpoints.
2. We split the 347 controls in the second time frame (2011-2013) into three groups of nearly equal sizes and equal numbers of events with respect to both the OS and rPFS endpoints.
3. We randomly combined the five datasets from step 1 with the data from the five experimental arms and the comparisons A vs. B, A vs. C and A vs. E were combined with one dataset each generated from step 2.

We analyzed the data using the eight trials and the five pseudo-trials. And then we repeated the random splitting and the data analysis 100 times and took the average of the results of these 100 splits. For each measure (i.e., R^2, STE, HR, Kendall's tau), the results among the 100 splits that is closest to the average was selected and we reported those as the estimates.

For the androgen deprivation therapy (ADT) subset analysis (A vs. B, A vs. D, and A vs. F), the 377 shared controls in the first time period were split into three groups with nearly equal sizes and equal number of events with respect to both the OS and rPFS endpoints. For the second time frame, the 347 controls were for A vs. B with no data splitting required.

For the ADT plus docetaxel comparisons (A vs. C, and A vs. E), the 724 shared controls were split into two groups between comparisons A vs. C and A vs. E with nearly equal sizes and equal number of events with respect to both the OS and rPFS endpoints.
